# Retraction

**DOI:** 10.1002/cam4.6941

**Published:** 2024-02-09

‘Long non‐coding RNA ARAP1‐AS1 promotes tumorigenesis and metastasis through facilitating proto‐oncogene c‐Myc translation via dissociating PSF/PTB dimer in cervical cancer’ by Yao Zhang, Dan Wu, Dian Wang, *Cancer Med* 2020, 9: 1855–1866. The above article, published online on 17 January 2020 in Wiley Online Library (https://onlinelibrary.wiley.com/doi/10.1002/cam4.2860) has been retracted by agreement between the authors, the journal's Editor‐in‐Chief, Stephen Tait, and John Wiley & Sons Ltd.

The retraction has been agreed upon after the authors contacted the journal and indicated substantial mistakes during figure compilation of this manuscript. Additionally, some image elements of the experimental data were published elsewhere in a different scientific context. Thus, the editors consider the conclusions of this article to be invalid.

